# Systemic depletion of WWP1 improves insulin sensitivity and lowers triglyceride content in the liver of obese mice

**DOI:** 10.1002/2211-5463.13610

**Published:** 2023-04-20

**Authors:** Yuka Nozaki, Masaki Kobayashi, Hiroki Wakasawa, Shunsuke Hoshino, Fumika Suwa, Yuko Ose, Ryoma Tagawa, Yoshikazu Higami

**Affiliations:** ^1^ Laboratory of Molecular Pathology and Metabolic Disease, Faculty of Pharmaceutical Science Tokyo University of Science Chiba Japan; ^2^ Department of Nutrition and Food Science, Graduate School of Humanities and Sciences Ochanomizu University Tokyo Japan; ^3^ Institute for Human Life Innovation Ochanomizu University Tokyo Japan

**Keywords:** adipose tissue, hepatic steatosis, obesity, WWP1

## Abstract

Obesity is a metabolic disorder associated with many diseases. WW domain‐containing E3 ubiquitin protein ligase 1 (WWP1) is a HECT‐type E3 ubiquitin ligase involved in several diseases. Recently, we found that the level of WWP1 is increased in white adipose tissue in a mouse model of obesity and that obese *Wwp1* knockout (KO) mice exhibit improved whole‐body glucose metabolism. Here, to determine which insulin‐sensitive tissues contribute to this phenotype, we investigated the levels of several insulin signaling markers in white adipose tissue, liver, and skeletal muscle of *Wwp1* KO mice, which were fed a normal or high‐fat diet and transiently treated with insulin. In obese *Wwp1* KO mice, phosphorylated Akt levels were increased in the liver but not in white adipose tissue or skeletal muscle. Moreover, the weight and triglyceride content of the liver of obese *Wwp1* KO mice were decreased. These results suggest that systemic deletion of WWP1 improves glucose metabolism via enhanced hepatic insulin signaling and suppressed hepatic fat accumulation. In summary, WWP1 participates in obesity‐related metabolic dysfunction and pathologies related to hepatic steatosis via suppressed insulin signaling.

## Graphical abstract

In obese WW domain‐containing E3 ubiquitin protein ligase 1 (*Wwp1*) KO mice, phosphorylated Akt levels were increased in the liver. Moreover, the weight and triglyceride content of the liver of obese *Wwp1* KO mice were decreased. These results suggest that systemic deletion of WWP1 improves glucose metabolism via enhanced hepatic insulin signaling and suppressed hepatic fat accumulation.

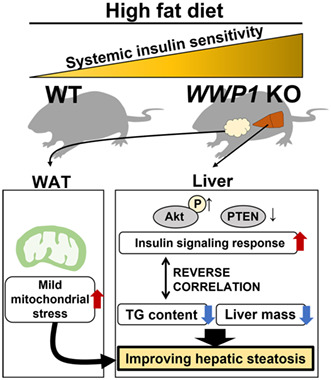

**Abbreviations**
ACC: acetylcholine‐gated chloride channel subunit
ACLY: ATP citrate synthase
AIP5: atrophin‐1‐interacting protein 5
CBB: Coomassie Brilliant Blue
eWAT: epididymal white adipose tissue
FASN: fatty acid synthase
GTT: glucose tolerance test
HECT: homologous to the E6‐AP carboxyl terminus
HFD: high‐fat diet
IRS: insulin receptor substrate
ITT: insulin tolerance test
NAFLD: nonalcoholic fatty liver disease
NASH: nonalcoholic steatohepatitis
ND: normal diet
PTEN: phosphatase and tensin homolog
sWAT: subcutaneous white adipose tissue
TIUL1: TGIF‐interacting ubiquitin ligase 1
WWP1: WW domain‐containing E3 ubiquitin protein ligase 1

Obesity is abnormal or excessive fat accumulation that causes metabolic disorders and contributes to the development of insulin resistance and type 2 diabetes through chronic inflammation or oxidative stress [1]. Since obesity‐related insulin resistance has become a major worldwide health problem, it is necessary to understand the molecular mechanisms underlying insulin resistance and establish preventive methods.

Insulin has important metabolic effects in insulin‐sensitive tissues, which include the liver, white adipose tissue (WAT), and skeletal muscle. Insulin stimulation suppresses glucose output in the liver and lipolysis in WAT. In addition, it enhances hepatic glycogen production and glucose uptake in WAT and skeletal muscle. Insulin resistance is characterized by a diminished response to insulin stimulation that results, in part, from disruption of the insulin signaling pathway [2].

In this pathway, the binding of insulin to the insulin receptor induces tyrosine phosphorylation of the insulin receptor substrate via autophosphorylation of the insulin receptor. This phosphorylation activates phosphatidylinositol 3‐kinase (PI3K), which converts phosphatidylinositol 3,4‐bisphosphate (PIP_2_) into phosphatidylinositol 3,4,5‐triphosphate (PIP_3_), a phospholipid recognized as the cellular second messenger. Phosphatase and tensin homolog (PTEN) is a negative regulator of PI3K [3, 4]. Akt (also known as protein kinase B) is phosphorylated and activated by PIP_3_, leading to induced translocation of glucose transporter 4 on the cell surface and glucose uptake in insulin‐sensitive tissues [5]. Therefore, the insulin signaling pathway is important for whole‐body glucose metabolism.

WW domain‐containing E3 ubiquitin protein ligase 1 (WWP1; also known as TIUL1 or AIP5) belongs to the HECT‐type E3 ubiquitin protein ligase family. WWP1 has a C2 domain at its N‐terminal, four WW domains in its central region and a HECT domain at its C‐terminal [6, 7]. The C2 domain determines subcellular localization of the molecule, while the WW domains bind to proline‐rich sequences (PY motif) of substrate proteins. Previous studies have indicated that WWP1 plays important roles in various pathologies such as cancers, infectious diseases, and neurological diseases [7].

We previously revealed that in a mouse model of obesity induced by a high‐fat diet (HFD), the expression level of WWP1 in WAT was elevated in a p53‐dependent manner [8]. Subsequently, to evaluate the involvement of WWP1 in metabolic regulation, we studied systemic *Wwp1* knockout (KO) mice and found that the HFD‐induced reduction in phosphorylation levels of Akt in WAT was enhanced in *Wwp1* KO mice. This result indicates that deletion of WWP1 exacerbates obesity‐related insulin resistance in WAT. However, in the same study, the insulin tolerance test (ITT) and glucose tolerance test (GTT) showed that insulin sensitivity and glucose tolerance were unexpectedly improved in *Wwp1* KO mice [9]. This discrepancy between insulin sensitivity in the whole body and insulin signal transduction in WAT implies that WWP1 deletion may also affect insulin signal transduction in other insulin‐sensitive tissues, including the liver and skeletal muscle. Thus, in this study, we investigated insulin signal transduction in insulin‐sensitive tissues in *Wwp1* KO mice transiently stimulated with insulin.

## Methods

### Animals

All animal experiments and protocols were conducted in accordance with the Fundamental Guidelines for Proper Conduct of Animal Experiments and Related Activities in Academic Research Institutions under the jurisdiction of the Ministry of Education, Culture, Sports, Science and Technology of Japan and were approved by the Ethics Review Committee for Animal Experimentation at Tokyo University of Science (approval numbers: Y20043 and Y21043). Mice with systemic KO of *Wwp1* (*Wwp1* ^−/−^ mice) and wild‐type (WT) *Wwp1* ^+/+^ mice were generated as shown in our previous report [10]. Genotyping of offspring was performed by PCR using KOD FX neo (Toyobo, Osaka, Japan) with the following primers: forward, 5′‐AGA GGC AAG AGA ATG GCG TCA AG‐3′; reverse, 5′‐GGA GGT GAA AGG GTT GGA AGA ATA C‐3′. Mice were maintained under specific‐pathogen‐free conditions at 23 °C, under a 12‐h light/dark cycle in the animal facility at the Faculty of Pharmaceutical Sciences, Tokyo University of Science. They had free access to water and were fed a Charles River Formula‐1 diet (21.9% crude protein, 5.4% crude fat and 2.9% crude fiber; Oriental Yeast, Tokyo, Japan). At 5 weeks old, WT and KO mice were divided into two groups: the normal diet (ND; Nosan, Yokohama, Japan) group or HFD group. The Charles River Formula‐1 diet and High‐Fat Diet 32 (25.5% crude protein, 32.0% crude lipid and 2.9% crude fiber; CREA, Tokyo, Japan) were fed as the ND and HFD, respectively. At 13–15 weeks old, mice were euthanized under isoflurane anesthesia (Mylan, Canonsburg, PA, USA) in a fed state, and their liver was collected for measuring the expression of WWP1 by immunoblot (Fig. 3A). At 23 weeks old, mice were fasted for 24 h and intravenously administrated with insulin (1 U per kg body weight) via the inferior vena cava by abdominal section under isoflurane anesthesia. After 10 min, they were euthanized and their epididymal and subcutaneous WAT depots and liver and quadriceps femoris muscle (skeletal muscle) were collected and weighed for immunoblot and for measuring the contents of triglyceride and liver weight. These tissues were immediately diced, frozen in liquid nitrogen, and stored at −80 °C.

### Immunoblotting

The preparation of WAT lysates and immunoblotting were performed according to our previously reported methods [11]. Briefly, WAT was lysed in SDS sample buffer [50 mm Tris–HCl (pH 6.8), 2% SDS, 3 m urea, 6% glycerol], boiled for 5 min, and sonicated. Lysates were subjected to SDS/PAGE (15 μg protein per well), and separated proteins were transferred to nitrocellulose membranes. Membranes were blocked with blocking solution [2.5% skim milk, 0.25% BSA in TTBS (25 mm Tris–HCl pH 7.4, 140 mm NaCl, 2.5 mm KCl, 0.1% Tween‐20)] for 1 h at room temperature and then probed with appropriate primary antibodies overnight at 4 °C. The anti‐WWP1 antibody was originally generated in our laboratory [8]. The anti‐phospho‐Akt (Ser473; #9271), anti‐Akt (#9272), and anti‐ACC (acetyl‐CoA carboxylase; #3662) were from Cell Signaling Technology (Danvers, MA, USA). The anti‐ACLY (ATP citrate lyase; #1699‐1) and anti‐phospho‐ACLY (#1822‐1) were from Epitomics—an Abcam Company (Cambridge, UK). The FASN (fatty acid synthase; #910963) was from BD Bioscience (Franklin Lakes, NJ, USA), the Lamin B1 (#PM064) was from MBL (Aichi, Japan), and the anti‐PTEN (#sc‐7974) was from Santa Cruz Biotechnology (Dallas, TX, USA). Subsequently, membranes were incubated with appropriate secondary antibodies [horseradish peroxidase‐conjugated F(ab’)2 fragment of goat anti‐mouse IgG or anti‐rabbit IgG was by Jackson ImmunoResearch (West Grove, PA, USA)] for 1 h at room temperature. Antibody‐bound proteins were visualized using ImmunoStar LD Reagent by Wako (Osaka, Japan) and a LAS3000 Image Analyzer was by Fujifilm (Tokyo, Japan), and data were analyzed using multigage software (GE Healthcare, Chicago, WI, USA) or imagej software from NIH (Bethesda, MD, USA). Specific signal intensities were normalized to those of Lamin B1 or Coomassie Brilliant Blue staining.

### GTT and ITT

GTT and ITT were performed in HFD‐fed *Wwp1* WT and KO mice of 13–15 weeks old. Prior to GTT and ITT, mice were fasted for 24 and 8 h, respectively. D‐glucose (1.0 kg per body weight, Wako) or insulin (1.0 U per kg body weight, Wako) were injected intraperitoneally for GTT and ITT, respectively. Next, serial blood sampling from the tail vein was performed at 0, 15, 30, 60, and 120 min after injection. Blood glucose levels were measured using an Accu‐Chek® Aviva blood glucose meter (Roche, Basel, Switzerland).

### Amount of triglyceride in liver

Collected liver pieces were homogenized with Solution I (chloroform : methanol = 1 : 1), followed by mixing with a quarter volume of 1 m NaCl. The homogenates were centrifuged for 10 min at 1100 ***g*** at 4 °C, and their lower layers were collected and dried up into lipid pellets. The pellets were dissolved in Solution II (tert‐butanol : methanol: triton‐114 = 3 : 1 : 1) as extracted triglyceride samples. The amount of triglyceride in samples was measured using LabAssay™ triglyceride (Wako).

### Measurement of glutathione concentrations

Total glutathione (tGSH [GSH + glutathione disulfide (GSSG)]) and GSSG concentrations were measured as previously reported [12]. WAT was homogenized in extraction buffer (0.1 m potassium phosphate buffer containing 5 mm EDTA (pH 7.5), 0.1% Triton X‐100, and 0.6% sulfosalicylic acid) and centrifuged at 4 °C for 10 min. Supernatants were used for the measurement of tissue GSH content with an Infinite F200 PRO microplate reader (Tecan, Männedorf, Switzerland). The rate of 5,5‐dithio‐bis‐(2‐nitrobenzoic) acid (DTNB) formation was calculated, and the concentrations of tGSH and GSSG in each sample were determined using linear regression, with reference to a standard curve. GSH concentration was calculated by subtracting GSSG concentration from tGSH concentration.

### Statistical analysis

Statistical significance was determined using the Student's *t*‐test to compare two groups or the Tukey–Kramer test to compare more than two groups after the assessment of significant differences by two‐way or three‐way analysis of variance. r software (R project for Statistical Computing) and/or BellCurve for Excel (Social Survey Research Information Co., Ltd., Tokyo, Japan) was used. Differences with *P* values < 0.05 were considered statistically significant.

## Results

### Deletion of WWP1 enhanced the insulin signaling response in liver from HFD‐fed mice

**Fig. 1:**
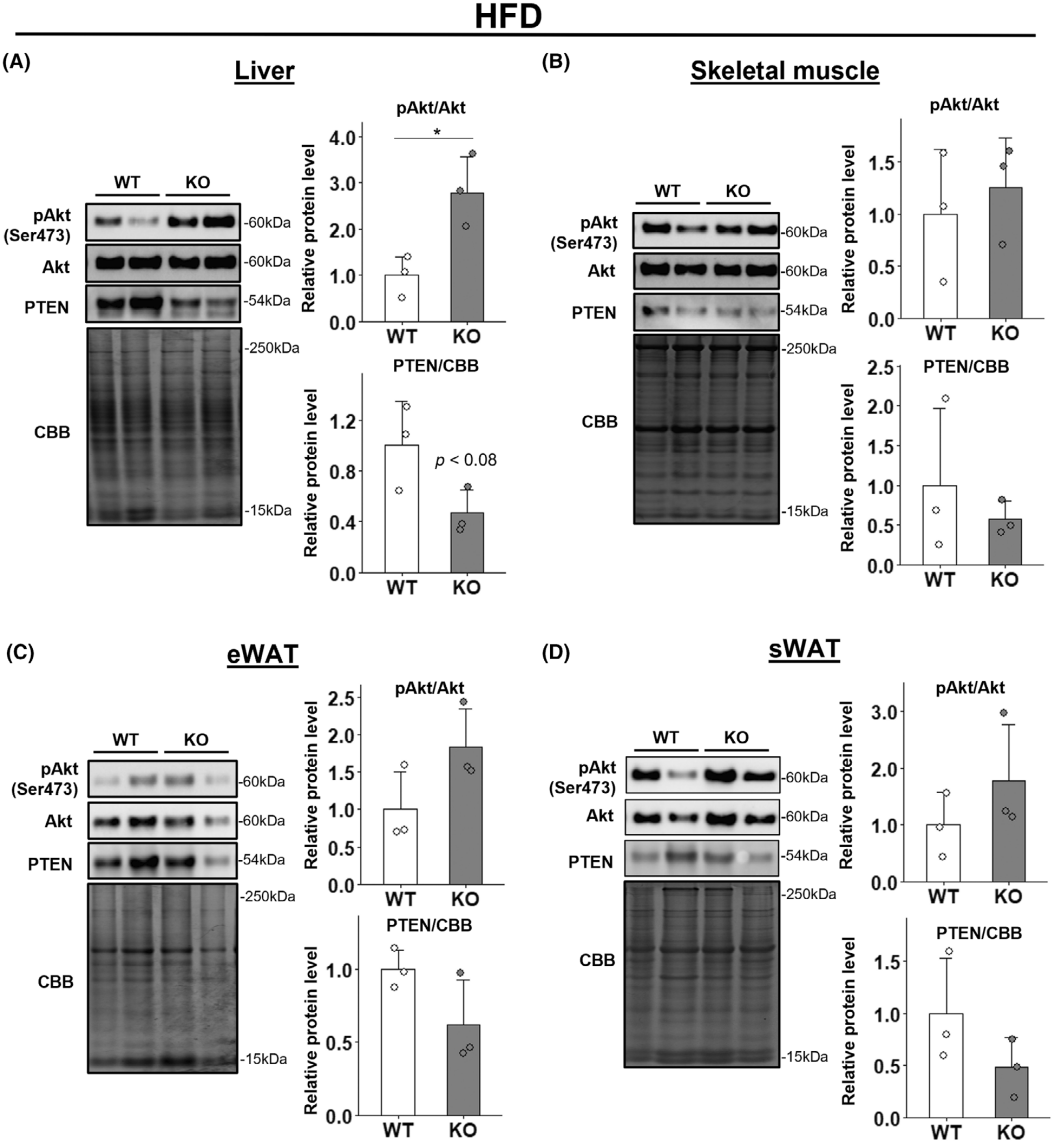
WWP1 KO increased expression of insulin‐signaling‐related proteins in liver derived from HFD‐fed mice. Male 23‐week‐old mice fed a HFD were treated with insulin by intravenous injection for 10 min. (A–D) Expression levels of insulin‐signaling‐related proteins in liver (A), skeletal muscle (B), epididymal WAT (C) and subcutaneous WAT (D) were obtained by immunoblotting (left, representative immunoblot data; right, quantitative values). Coomassie Brilliant Blue stain was used as a loading control. Circles indicate values of individual mice. Data are mean ± SD (*n* = 3–5 per group). Differences between each value were analyzed by Student's *t*‐test (*P < 0.05).

**Fig. 2:**
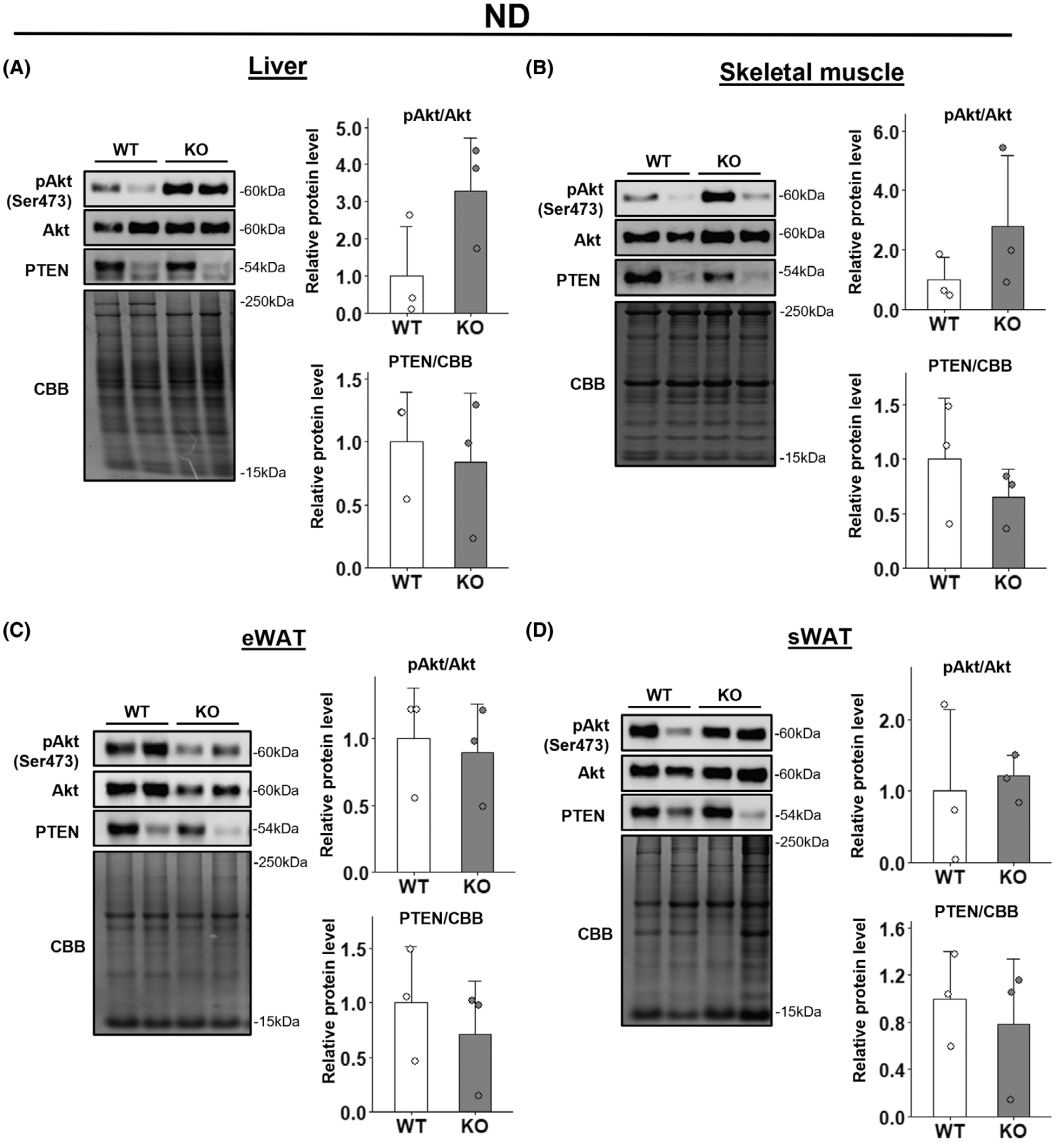
WWP1 KO had no effect on expression of insulin‐signaling‐related proteins in ND‐fed mice. Male 23‐week‐old mice fed a ND were treated with insulin by intravenous injection for 10 min. (A–D) Expression levels of insulin‐signaling‐related proteins in liver (A), skeletal muscle (B), epididymal WAT (C) and subcutaneous WAT (D) were obtained by immunoblotting (left, representative immunoblot data; right, quantitative values). Coomassie Brilliant Blue stain was used as a loading control. Circles indicate values of individual mice. Data are mean ± SD (*n* = 3–5 per group). Differences between each value were analyzed by Student's *t*‐test.

We initially performed GTT and ITT and confirmed that insulin sensitivity was improved in HFD‐fed *Wwp1* KO mice, which is consistent with our published results (Fig. S1B,D) [9]. By contrast, these changes were not observed in ND‐fed mice (Fig. S1A,C). To evaluate the insulin signaling response in insulin‐sensitive tissues (including epididymal and subcutaneous WAT, skeletal muscles and liver), we analyzed insulin‐stimulated changes in levels of total and phosphorylated Akt (Akt and pAkt) and PTEN, which are insulin signaling‐related proteins, in each tissue in *Wwp1* KO mice. The results showed that in the liver of HFD‐fed mice, WWP1 deletion significantly increased the ratio of pAkt/Akt protein levels and, despite no significance, decreased PTEN protein levels (Fig. 1A). In the WAT and skeletal muscle of HFD‐fed mice, however, WWP1 deletion did not affect levels of these proteins (Fig. 1B–D). Moreover, in ND‐fed mice, WWP1 deletion did not significantly affect the ratio of pAkt/Akt levels and PTEN levels in any tissue (Fig. 2). These results suggest that the improvement in insulin sensitivity in *Wwp1* KO mice results from an enhanced insulin signaling response mainly in the liver.

### Deletion of WWP1 decreased the weight and triglyceride content of the liver in HFD‐fed mice

**Fig. 3:**
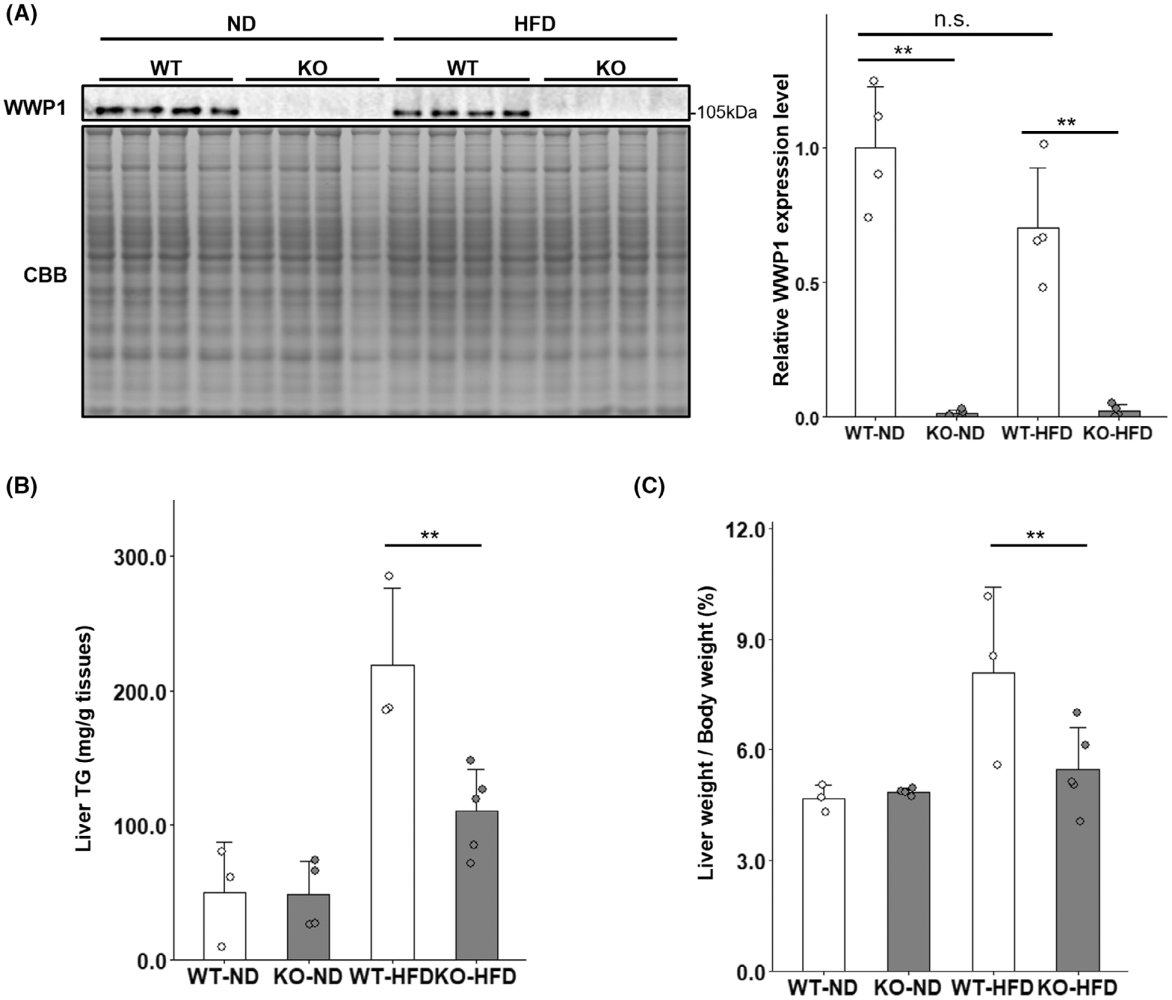
WWP1 KO decreased the weight and triglyceride content of the liver in HFD‐fed mice. (A) Expression levels of WWP1 in ND‐fed and HFD‐fed WT and KO mice of 13–15 weeks of age (left, representative immunoblot data; right, quantitative values). Coomassie Brilliant Blue stain was used as a loading control. (B) Triglyceride content of liver in 23‐week‐old mice from all groups. (C) Liver weight in 23‐week‐old mice from all groups treated with insulin by intravenous injection for 10 min. Circles indicate values of individual mice. Bar represents mean ± SD (*n* = 3–5 per group). Differences between each value were analyzed by the Tukey–Kramer test (**P < 0.01).

Protein levels of WWP1 in WT mice were not different between ND‐fed and HFD‐fed mice of 13–15 weeks of age in the liver (Fig. 3A). Hepatic insulin sensitivity is reportedly inversely correlated with triglyceride content in nondiabetic obese patients [13]; therefore, we measured the hepatic triglyceride content in all groups of mice of 23 weeks of age. We showed that HFD‐induced increases in hepatic triglyceride content were significantly suppressed in *Wwp1* KO mice (Fig. 3B). In addition, liver weight was also decreased in HFD‐fed *Wwp1* KO mice, which correlates with the decrease in triglyceride content (Fig. 3C). *De novo* lipogenesis is important for regulation of hepatic triglyceride levels [14]. To assess the contribution of WWP1 to lipogenesis in the liver, we examined the expression levels of ACC, FASN, and phosphorylated ACLY, which are important enzymes in *de novo* fatty acid synthesis. The results showed that expression of these proteins was not significantly different in any of the groups (Fig. S2).

## Discussion

Our previous report demonstrated that obese systemic *Wwp1* KO mice exhibit improved whole‐body insulin sensitivity (Fig. S1 and [9]). In this study, we focused on insulin‐sensitive tissues and investigated the insulin signaling pathway to assess the role of WWP1 in whole‐body glucose metabolism. We found that WWP1 deletion in HFD‐fed mice enhanced insulin signaling (pAkt/Akt rate) in the liver (Fig. 1A) but not in other insulin‐sensitive tissues (Fig. 1B–D). This enhanced insulin signaling in the liver by WWP1 deletion probably contributes to improved whole‐body glucose metabolism in *Wwp1* KO obese mice. In addition, WWP1 deletion in HFD‐fed mice slightly reduced PTEN protein levels in the liver, suggesting that WWP1 may positively regulate PTEN (Fig. 1D). By contrast, several studies have shown that WWP1 negatively regulates PTEN in cancers. In studies by Lee et al., WWP1 ubiquitinated PTEN and inhibited its dimerization and translocation to the cellular membrane, resulting in the inactivation of PTEN [15, 16]. Furthermore, total protein levels of PTEN were not altered in embryonic fibroblasts of *Wwp1* KO mice in one of these studies [15]. Considering that the HFD did not affect WWP1 levels in the liver in our study (Fig. 3A), the discrepancy between our results and these findings implies that WWP1 deletion reduces PTEN protein levels in obese liver in an indirect manner, for example, by the influence of other organs. Taken together, although the mechanism remains unclear, WWP1 plays a downregulatory role in hepatic insulin signal transduction via stabilizing PTEN, at least in obese mice.

Although HFD did not alter WWP1 expression levels in the liver (Fig. 3A), we previously reported that WWP1 expression was increased by HFD in WAT in a p53‐dependent manner [8]. Considering the evidence that obesity upregulated p53 expression not only in WAT but also in liver [17], it is conceivable that the regulation of HFD‐induced WWP1 expression in liver is different from that in WAT, which is dependent on p53. Moreover, it was reported that transforming growth factor β (TGFβ) [18] and tumor necrosis factor α (TNFα) [19] stimulate the transcription of WWP1 gene via an unknown mechanism. Several micro‐RNAs have been also found to regulate the expression of WWP1 [20]. Despite no direct evidence, these regulators may contribute to the regulation of WWP1 expression in obese liver.

WWP1 deletion reduced increases in hepatic triglyceride content normally associated with a HFD (Fig. 3B). We previously demonstrated that WWP1 plays a defensive role against mitochondrial oxidative stress in adipocytes and WAT [8, 9]. Hence, the WAT of *Wwp1* KO mice is more vulnerable to mitochondrial oxidative stress than that of WT mice. In contrast, while WWP1 deficiency slightly reduced the total glutathione concentration with no significant in liver, the GSH/GSSG ratio, a marker of antioxidative capacity, did not change, unlike WAT (Fig. S3). Mild‐to‐moderate mitochondrial dysfunction or stress responses in adipocytes prevent obesity‐induced hepatic steatosis [21]. For example, Yang et al. [22] have shown that adipose‐specific deletion of fumarate hydrase, an integral Krebs cycle enzyme, provokes mitochondrial stress and suppresses hepatic steatosis. Systemic deletion of caseinolytic mitochondrial matrix peptidase proteolytic subunit (ClpP), a mitochondrial matrix protease responsible for quality control of mitochondrial proteins, also reportedly exerts similar effects [23]. These reports suggest that reductions in the weight and triglyceride content of the liver in obese *Wwp1* KO mice may result from moderate mitochondrial oxidative stress in adipocytes. Moreover, the above‐mentioned mouse models of mitochondrial oxidative stress exhibited improved glucose tolerance [22, 23]. Thus, although activation of insulin signal transduction in the liver is a main mechanism by which insulin sensitivity is enhanced in *Wwp1* KO mice, mitochondrial oxidative stress in adipocytes also plays a role.

In addition to the extrahepatic influence, Korenblat et al. [13] raise the possibility that decreased hepatic triglyceride content is associated with an enhanced insulin signaling response in the liver; in this study, hepatic triglyceride content was inversely correlated with hepatic insulin sensitivity in obese nondiabetic patients, despite an unproved cause‐and‐effect relationship. This report also found that hepatic triglyceride content was directly correlated with basal plasma insulin concentration in obese nondiabetic patients, in agreement with our previous finding of decreased plasma insulin levels in HFD‐fed *Wwp1* KO mice [8]. Collectively, decreased weight and triglyceride content in the liver of *Wwp1* KO mice could be explained by two mechanisms: one is oxidative‐stress‐induced mitochondrial dysfunction in WAT, and the other is enhanced insulin signal transduction in the liver. Clarification of which mechanism is correct requires analysis of adipose‐specific or liver‐specific *Wwp1* KO mice.

In the present study, obese *Wwp1* KO mice displayed an enhanced hepatic insulin signaling response. In obese mice, WWP1 deletion also reduced the weight and triglyceride content of the liver. Dysregulation of hepatic triglyceride content is closely linked to nonalcoholic fatty liver disease (NAFLD)/nonalcoholic steatohepatitis (NASH). NAFLD/NASH is an obesity‐associated risk factor for serious liver diseases, including cirrhosis and hepatocellular carcinoma [24]. Zhang et al. [25] have reported that the expression of WWP1 is upregulated in human hepatocellular carcinoma and is highly correlated with its poor outcome. Therefore, further analysis of WWP1 will improve understanding of not only obesity‐related metabolic dysfunction but also hepatic steatosis‐related pathologies, such as cirrhosis and hepatocellular carcinoma.

## Conflict of interest

The authors declare no conflict of interest.

## Author contributions

MK, RT, and YH supervised the study and designed the experiments; YN, HW, SH, FS, and YO performed the experiments; YN and HW analyzed the data; YN wrote the manuscript; and MK, RT, and YH made manuscript revisions.

## Supporting information


**Fig. S1.** WWP1 KO improved glucose metabolism in HFD‐fed mice. Blood glucose levels during intraperitoneal GTT (A, B) or ITT (C, D) studies in all groups of mice of 13–15 weeks of age. Each area under the curve (AUC) is calculated and shown. The quantitative values are mean ± SD (n = 5–8 per group). Differences between each value were analyzed by Student's t‐test. (##, *P* < 0.01, ###, *P* < 0.001).

**Fig. S2.** WWP1 KO does not affect lipogenesis‐related protein expression in the liver of HFD‐fed mice. Male 23‐week‐old mice fed a HFD were treated with insulin by intravenous injection for 10 min. (A, C) Expression levels of lipogenesis‐related proteins in liver from ND‐fed or HFD‐fed mice were obtained by immunoblotting. (B, D) The quantitative values of ACC, FASN, and phosphorylated ACLY normalized by total ACLY. Lamin B was used as a loading control. Circles indicate values of individual mice. Data are mean ± SD (n = 3–5 per group). Differences between each value were analyzed by Student's t‐test.

**Fig. S3.** WWP1 KO does not affect antioxidative reaction in the liver of HFD‐fed mice. (A‐B) Glutathione concentrations in liver from ND‐fed and HFD‐fed WT and KO mice of 23 weeks of age with insulin by intravenous injection for 10 min were measured spectrophotometrically at 412 nm. (A) Total GSH and (B) GSH/GSSG ratio. Circles indicate values of individual mice. Bar represents mean ± SD (n = 3–5 per group). Differences between each value were analyzed by the Tukey–Kramer test.

## Data Availability

The data that support the findings of this study are available in the figures and the supporting information.
