# Supplementary figures and images for: Systemic depletion of WWP1 improves insulin sensitivity and lowers triglyceride content in the liver of obese mice
Source: FEBS Open Bio. 2023 Apr 20;13(6):1086–94. doi: 10.1002/2211-5463.13610 (PMC10240335; doi:10.1002/2211-5463.13610)

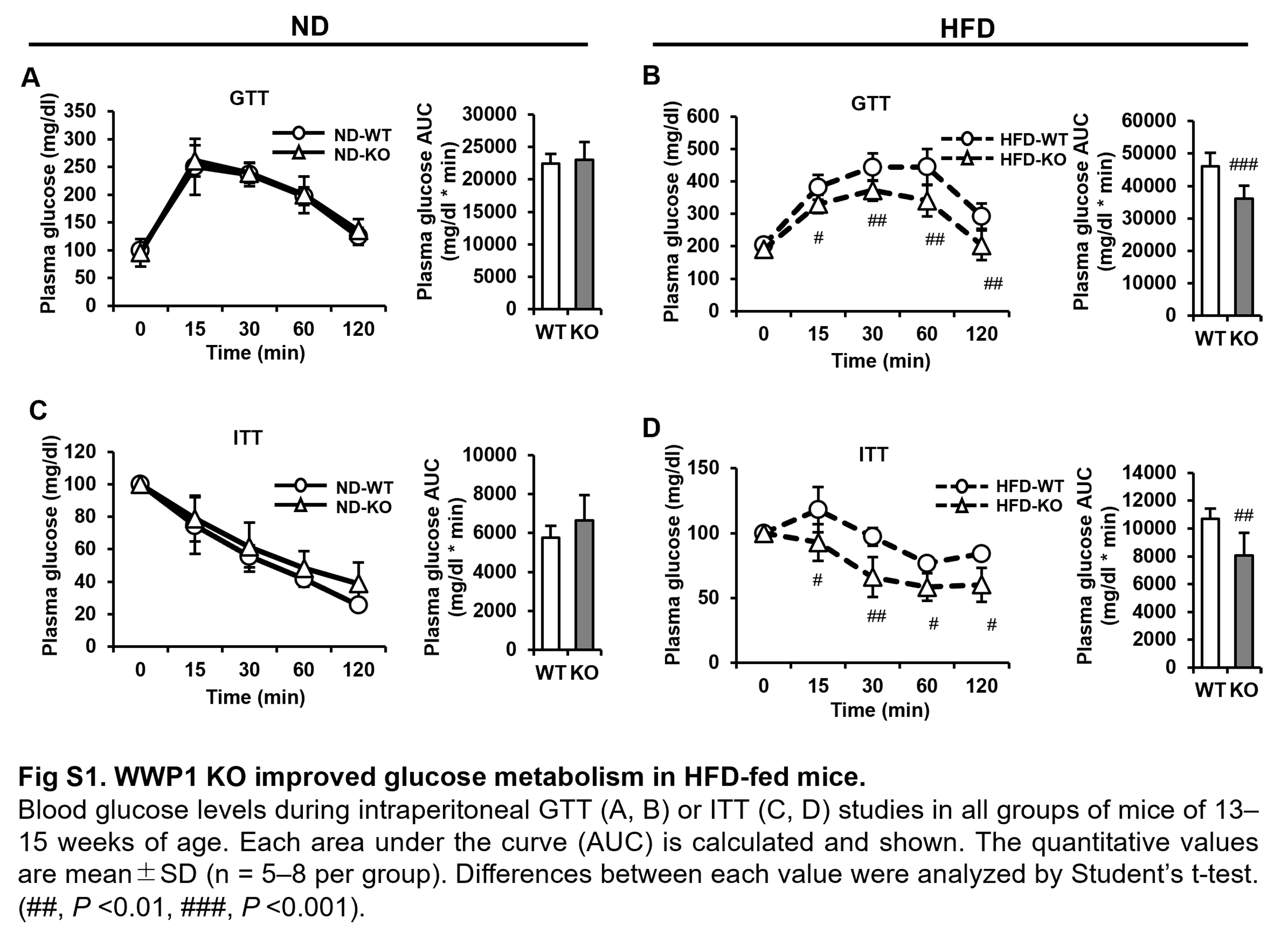

Supplement: Supplementary file 1 — Fig. S1. WWP1 KO improved glucose metabolism in HFD‐fed mice. Blood glucose levels during intraperitoneal GTT (A, B) or ITT (C, D) studies in all groups of mice of 13–15 weeks of age. Each area under the curve (AUC) is calculated and shown. The quantitative values are mean ± SD (n = 5–8 per group). Differences between each value were analyzed by Student's t‐test. (##, P < 0.01, ###, P < 0.001). [file FEB4-13-1086-s002.tif]

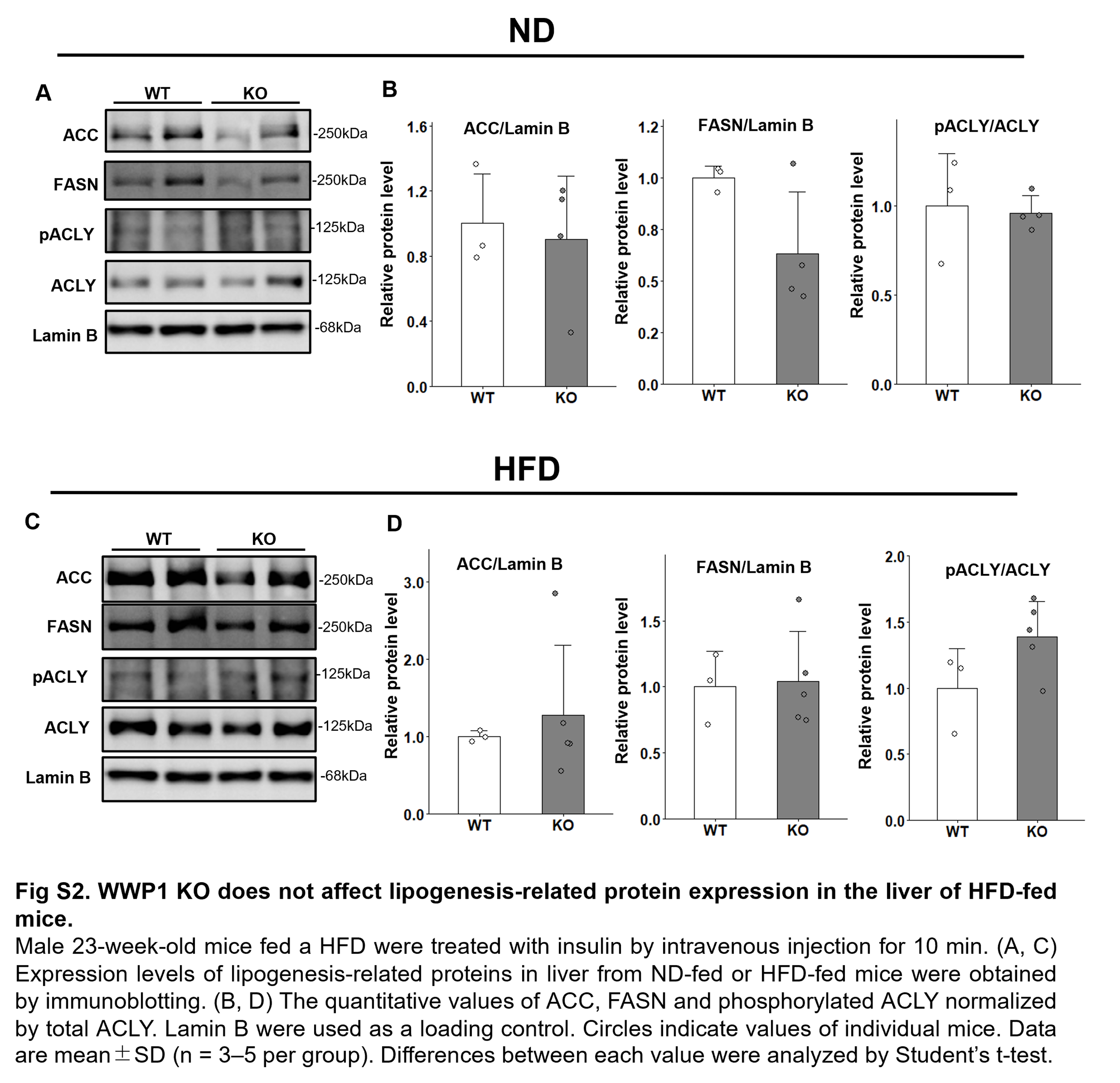

Supplement: Supplementary file 2 — Fig. S2. WWP1 KO does not affect lipogenesis‐related protein expression in the liver of HFD‐fed mice. Male 23‐week‐old mice fed a HFD were treated with insulin by intravenous injection for 10 min. (A, C) Expression levels of lipogenesis‐related proteins in liver from ND‐fed or HFD‐fed mice were obtained by immunoblotting. (B, D) The quantitative values of ACC, FASN, and phosphorylated ACLY normalized by total ACLY. Lamin B was used as a loading control. Circles indicate values of individual mice. Data are mean ± SD (n = 3–5 per group). Differences between each value were analyzed by Student's t‐test. [file FEB4-13-1086-s001.tif]

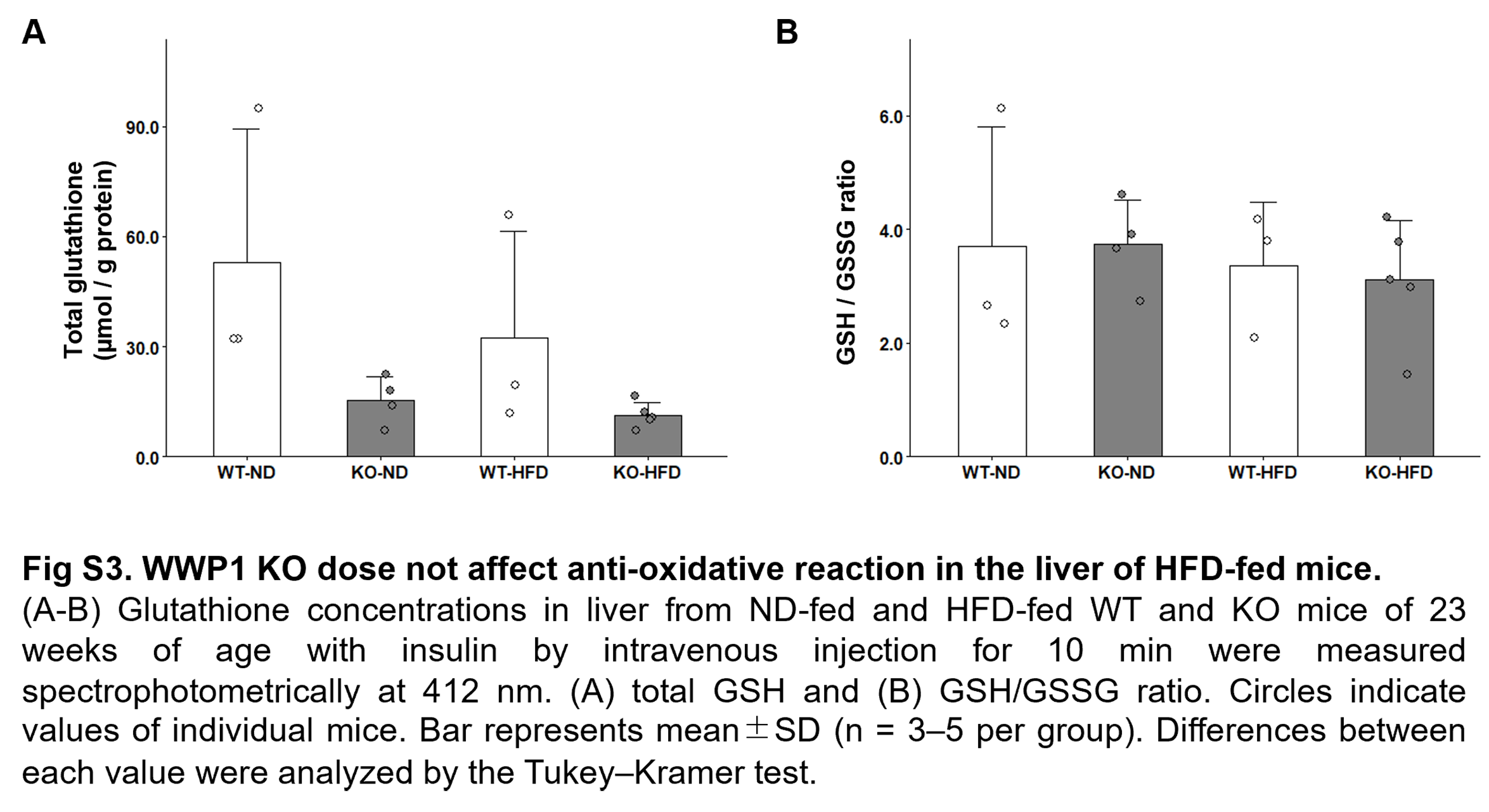

Supplement: Supplementary file 3 — Fig. S3. WWP1 KO does not affect antioxidative reaction in the liver of HFD‐fed mice. (A‐B) Glutathione concentrations in liver from ND‐fed and HFD‐fed WT and KO mice of 23 weeks of age with insulin by intravenous injection for 10 min were measured spectrophotometrically at 412 nm. (A) Total GSH and (B) GSH/GSSG ratio. Circles indicate values of individual mice. Bar represents mean ± SD (n = 3–5 per group). Differences between each value were analyzed by the Tukey–Kramer test. [file FEB4-13-1086-s003.tif]
